# Impairment of lung volume perception and breathing control in hypermobile Ehlers-Danlos syndrome

**DOI:** 10.1038/s41598-024-58890-2

**Published:** 2024-04-06

**Authors:** Adrien Hakimi, Cyrille Bergoin, Anna De Jesus, Eric Hermand, Claudine Fabre, Patrick Mucci

**Affiliations:** 1 Univ. Lille, Univ. Artois, Univ. Littoral Côte d’Opale, ULR 7369 - URePSSS - Unité de Recherche Pluridisciplinaire Sport Santé Société, 59000 Lille, France; 2 Clinique de la Mitterie, Lomme, France; 3 Cabinet de Pneumologie, Tourcoing, France; 4 Université Sorbonne Paris Nord, UMR INSERM U1272 Hypoxie & Poumon, Bobigny, France; 5 URePSSS, Eurasport, 413, Avenue Eugène Avinée, 59120 Loos, France

**Keywords:** Musculoskeletal system, Medical research, Physiology, Respiration, Respiratory signs and symptoms

## Abstract

Breathing difficulties and exertional dyspnea are frequently reported in hypermobile Ehlers-Danlos syndrome (hEDS); however, they are not clearly explained. An impaired proprioception or the addition of a cognitive task could influence ventilatory control. How can the perception of lung volume be measured? Is lung volume perception impaired in hEDS patients? Is the breathing control impaired during a cognitive task in hEDS patients? A device was developed to assess the accuracy of lung volume perception in patients with hEDS and matched control subjects. In the second step, ventilation was recorded in both groups with and without a cognitive task. Two groups of 19 subjects were included. The accuracy of lung volume perception was significantly (*P* < 0.01) lower at 30% of inspired vital capacity in patients with hEDS in comparison to the control group, and they showed erratic ventilation (based on spatial and temporal criteria) when performing a cognitive task. These data support the influence of the proprioceptive deficit on ventilatory control in hEDS patients. These elements may help to understand the respiratory manifestations found in hEDS. Future research should focus on this relationship between lung volume perception and ventilation, and could contribute to our understanding of other pathologies or exercise physiology.

*Trial registration number*: ClinicalTrials.gov, NCT05000151.

## Introduction

Ehlers-Danlos syndromes (EDSs) are part of the inherited connective tissue disorders and are mainly characterized by joint hypermobility, skin hyperextensibility, and tissue fragility^1^. These syndromes are classified into 13 subtypes and present great clinical and genetic heterogeneity^1^. The most common is the hypermobile Ehlers-Danlos syndrome (hEDS), whose diagnosis remains clinical^2^. A wide variety of clinical manifestations are present in hEDS such as pain, fatigue, dysautonomia, gastrointestinal disorders, anxiety, and respiratory symptoms^3–5^.

Breathing difficulties and exertional dyspnea are frequently reported^3,6–9^, but few studies have been conducted on lung function in patients with hEDS and tend to support normal lung function in hEDS^3,9–11^. The respiratory symptoms of hEDS are still unclear and raise many questions^6^. However, structural changes in the tissues involved in ventilation, related to the collagen or extracellular matrix abnormalities found in EDS, could lead to changes in ventilatory kinetics. Many hypotheses have been proposed, such as an alteration of diaphragm dynamics due to tissue hyperextensibility^5,8^, an increase in lung compliance^9,12^, or involvement of the reduced inspiratory muscle strength^11^. Another hypothesis is impaired signals from proprioceptive receptors, which could explain some respiratory impairments^8^. Proprioception is the conscious or unconscious perception of the position and movement of the different parts of the body^13^. It was demonstrated that patients with hEDS have proprioceptive impairments^14–16^. Breathing involves a large number of structures generating proprioceptive inputs (rib cage joints, respiratory muscles, etc.)^17^. However, although the control of ventilation is mainly regulated by chemoreceptors, the influence of proprioceptive inputs on this control remains relatively unexplored.

On the one hand we hypothesize that in patients with hEDS, the alteration of the proprioceptive inputs from the musculoskeletal system involved in ventilation could induce an impairment of the perception of lung volume and, thus, of the ventilatory control and could then explain in part some respiratory symptoms. To our knowledge, there is currently no validated tool to assess the accuracy of lung volume perception, which would reflect the proprioceptive sensitivity of the musculoskeletal system involved in ventilation. A simple and non-invasive tool was therefore designed to study differences between hEDS patients and healthy subjects.

On the other hand, it was demonstrated that hEDS patients present greater difficulty than healthy subjects in controlling their balance or walking in dual-task assessments^18,19^. It is known that cognitive tasks can affect ventilation in healthy people under different conditions^20^. We can, therefore, hypothesize that in patients with hEDS, cognitive tasks may increase the difficulty of controlling breathing.

This study had three objectives: (1) design a tool for the measure of lung volume perception; (2) investigate whether lung volume perception is impaired in hEDS patients; (3) investigate whether the control of breathing during a cognitive task is impaired in hEDS patients.

## Methods

### Participants

Participants were enrolled into two groups: a group of patients with hEDS and a group of matched healthy individuals. The inclusion criteria for the hEDS group were understanding the French language, being older than 18 years old, and having been diagnosed with hEDS according to the 2017 criteria^1^. The exclusion criteria for the hEDS patients were a known ongoing pregnancy, having a respiratory comorbidity independent of those linked to hEDS (other than asthma, hyperventilation, or dyspnea), or having already participated in a rehabilitation program. For each patient, a subject from the control group was matched for sex, age (± 5 years), and body mass index (BMI) (± 2.5 kg.m^−2^). The participants in the control group were recruited from volunteer employees of the Clinique de la Mitterie (Lomme, France). The exclusion criteria for the control group were a known respiratory or proprioceptive pathology, hypermobility according to the Beighton score (≥ 5 under 50 years old or ≥ 4 over 50 years old), or a known ongoing pregnancy.

All participants were volunteers and gave their written informed consent. This study was approved by a national ethics committee (CPP Ouest II, 2021/54) and was registered on ClinicalTrials.gov (NCT05000151). All experiments were performed in accordance with the Declaration of Helsinki.

After giving their informed consent, participants first completed the questionnaire before the investigator proceeded to a measurement of the Beighton score, followed by spirometry measurements, perception of the lung volume, and impact of a cognitive task on ventilation. All measurements were conducted during the same session and by the same operator.

### General measurements

Participants completed a questionnaire including anthropometric data, occupation, inclusion and exclusion criteria, smoking status, and some questions on the subject’s feeling of skill in lung volume perception. Patients also completed a section on their diagnosis.

The questions about the subject’s feeling of skill in lung volume perception were: “Do you practice a hobby, physical activity or sport that requires good breathing control?” (Yes/No); “If yes, which one?” (Free answer); “Would you be able to estimate the quantity/volume of air entering your lungs?” (Yes/No) and “Circle the number that best describes your capacity/ability to perceive the quantity/volume of air entering your lungs.” (0–10 scale with 0 = “Not skilled at all. Unable to perceive.” and 10 = “Extremely skillful. Very good perception.”).

Hypermobility was assessed in all participants using the Beighton score^21^. Hypermobility was defined as a score of ≥ 5 for subjects under 50 years of age and ≥ 4 for subjects over 50 years of age^1^.

The Marshall’s questionnaire^22^ was used to look for any differences in physical activity levels between the groups that might influence the results. This questionnaire assesses the number of times the individual performs 20 min of intense physical activity or 30 min of moderate physical activity per week, providing a score from 0 to 8 with a threshold set at 4 (0–3: insufficiently active; 4–8: sufficiently active).

Inspiratory vital capacity (IVC), forced vital capacity (FVC), and forced expiratory volume in one second (FEV_1_) were measured on a calibrated computerized spirometer (BodyBox 5500, Medisoft, Belgium), according to the current recommendations^23^.

### Lung volume perception

To our knowledge, there is no validated tool to assess lung volume perception, whereas methods exist to assess limb proprioception. As an indicator of articular proprioception, the joint position sense is assessed actively or passively^13^. The principle is to define a target joint amplitude, get the subject to perceive this amplitude actively or passively, and then ask the subject to reproduce (actively) or recognize (passively) the target amplitude. The difference between the achieved amplitude and the target amplitude is used as the criterion for the accuracy of the joint position sense. By transposition to the assessment of lung volume perception, a target lung volume (inspired air volume) is defined, and the subject is asked to reproduce this volume. The difference between the volume achieved and the target inspiratory volume is used as a criterion for the accuracy of lung volume perception and would reflect the proprioceptive sensitivity of the musculoskeletal system involved in ventilation.

The device for measuring lung volume perception had to display visual feedback in real time in order for the participant to adjust the inspiratory maneuver to reach the target volume in the first phase of the test. This device was composed of a mouthpiece (22M, Intersurgical, France), a single-use electrostatic filter (1420/01 or 1420/03, Medguard, Ireland), a 40.5 cm long and 22 mm diameter connecting tube, a flowmeter (SFM3000, Sensirion, Switzerland), an electronic acquisition card (UNO R3, Elegoo, China) with a 20 Hz sampling frequency, and an LCD screen for visual feedback. This device was calibrated with a 3 L syringe before each test (the same used for spirometer calibration) until an average of 6 measurements between 2985 and 3015 mL was obtained in accordance with the American Thoracic Society’s recommendations^23^. The program used for measurements was created with the software Arduino IDE Version 1.8.13 (Arduino.cc) and is based on the SFM3000Wedo library available online^24^. The device was positioned on a height-adjustable table so that the tube connecting the flowmeter to the patient’s mouth was horizontal in a standardized position. Subjects wore a nose clip to prevent nasal breathing and noise-canceling earbuds to cancel noise distraction.

First, an inspiratory vital capacity was measured with the device described above (IVC with flowmeter; IVC_fm_). Then, three target inspiratory volumes (IV) were defined according to IVC_fm_. An initial familiarization IV at 40% of IVC_fm_ (IV_40_) allowed the patient to become familiar with the procedure, followed by two IVs at 30% (IV_30_) and 50% (IV_50_) of the IVC_fm_ used to measure accuracy at different levels of structural stretch. The applied order (IV_40_, then IV_30_, then IV_50_) remained the same for all subjects. A break of one to two minutes was taken between each IV. The instruction for the participants was, after emptying their lungs, to inhale a volume as close as possible to these IVs. Participants were asked to empty their lungs before inhaling. The current IV was written on a board visible to the subject. The subject had three training sessions per IV, during which the volume was displayed in real time on an LCD screen. During training sessions, the subject was asked to reproduce the target volume and to focus on the perceived feelings in order to reproduce it afterward without a screen. The subject then performed three trials without visual feedback. The achieved volume was verbally indicated after each blind trial. Verbal instructions to the subjects were standardized.

**Figure 1:**
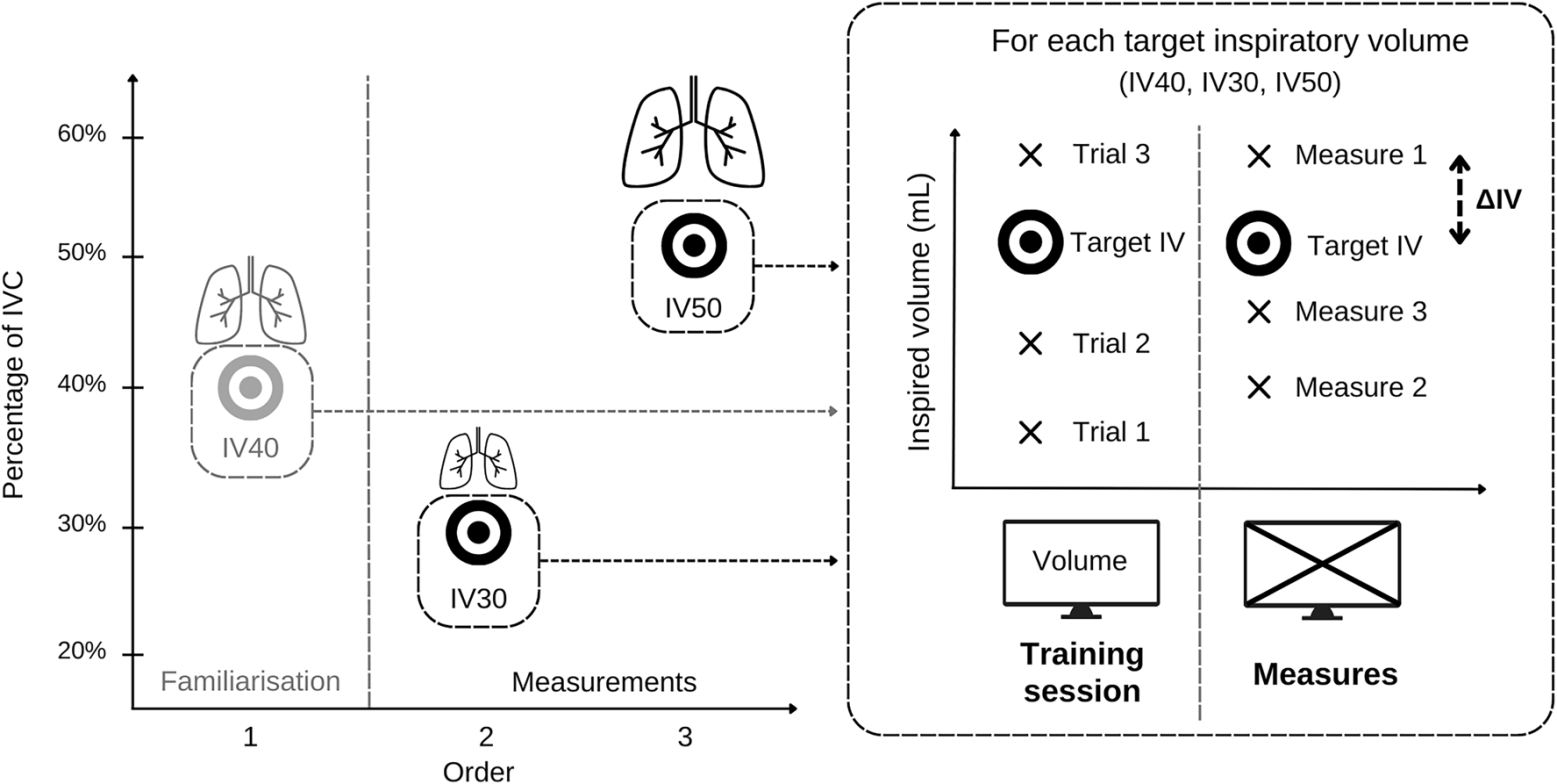
Schematic representation of the lung volume perception test design. On the left side is the overall process with the three target inspiratory volumes; on the right side is the process for each target inspiratory volume with the training session (visual feedback) and the measurements (no visual feedback). *ΔIV* absolute difference between the measure and the IV, *IVC* inspired ventilatory capacity; and *IV* target inspiratory volume.

The absolute difference between each trial and the IV (ΔIV) was calculated in milliliters and as a percentage of the IV. These values were used to calculate the average ΔIV for IV_30_ and IV_50_. A schematic representation of this design is presented in Fig. 1.

### Ventilatory response to cognitive task

The objective of this last measurement was to assess the influence of a cognitive task on the ventilatory control in hEDS patients versus healthy subjects. Wearing earbuds and nose clips, subjects first breathed freely through the same device one minute before, immediately afterward, and for one minute, during a cognitive task. The cognitive task was the Trail making test part B^25^, consisting of connecting, as quickly and accurately as possible, in ascending order circles containing numbers and letters, alternating one number and one letter each time. This test was used as a simple, standardized, non-verbal cognitive task requiring little movement. As recommended, an example was shown to the subject before the start of the two periods. As the first experimental condition was in a resting condition (subjects sitting on a chair), there was no randomization of the order of conditions between subjects. The verbal instructions given to the participants were standardized.

In order to analyze the variability of ventilation, each inspiratory peak was identified. Only respiratory cycles longer than 0.1 s and larger than 100 mL were taken into account^26^. This made it possible to calculate the time intervals between two inspiratory peaks (PP) and the tidal volume (V_T_).

Temporal variations were measured by breathing frequency (f_B_), standard deviation of PP (PP_SD_), or root mean square of PP (RMSSD). Spatial variations were measured by the mean V_T_ and the standard deviation of this volume (V_T,SD_). Minute ventilation (V̇_E_) was used as a mixed index (temporal and spatial). The ventilatory coefficients of variability for both temporal (CoefVar_t) and spatial domains (CoefVar_V) were calculated as follows:

$$CoefVar\_t = \frac{{PP_{SD} }}{{PP_{mean} }};\quad CoefVar\_V = \frac{{V_{T,SD} }}{{V_{T,mean} }}$$

### Statistical analysis

Data are described with mean and standard deviation (mean ± SD). Statistical analysis was performed on SigmaStat Version 3.5 (Systat Software Inc., San Jose, USA). Before each test, the normality of the data distribution and equality of variances were tested. Differences between the two groups were tested with a paired *t*-test or a Wilcoxon signed-rank test. Differences were considered as significant for a *P* value < 0.05.

## Results

### Participants characteristics

Twenty patients were recruited in the hEDS group. One could not be matched due to a high BMI (42 kg.m^−2^), for which we could not find a matching control subject. This participant was therefore excluded from the analysis, and 19 control subjects were included. Each group consisted of 18 women and one man. Anthropometric data, Marshall and Beighton scores, and general information are presented in Table 1. There was no significant difference between the two groups for age, height, weight, BMI, and Marshall score, contrary to the Beighton score (*P* < 0.001). Four patients reported asthma, one of whom also reported hyperventilation.

**Table 1:** Subjects’ characteristics.

	hEDS (n = 19)	Control (n = 19)
Age (years)	33 ± 11	33 ± 9
Height (cm)	165 ± 6	166 ± 7
Weight (kg)	68 ± 13	69 ± 12
BMI (kg.m^-2^)	25.0 ± 4.8	25.1 ± 4.3
Marshall score	3.8 ± 2.5	3.7 ± 1.8
Sufficiently active subjects	n = 8 (42%)	n = 9 (47%)
Beighton score	5.5 ± 1.5	2.0 ± 1.3***
Active smoking	n = 3 (16%)	n = 1 (5%)
Professional status		
Professionally active	n = 13 (68%)	n = 18 (95%)
Student	n = 3 (16%)	n = 1 (5%)
Unemployed	n = 2 (10%)	
Invalided	n = 1 (5%)	
Diagnosis date		
< 1 year	n = 14 (74%)	
1–7 years	n = 5 (26%)	
IVC (mL)	3506 ± 871	3954 ± 395
FVC (mL)	3896 ± 678	4070 ± 425
FEV_1_ (L)	3.167 ± 0.588	3.292 ± 0.312

Data are presented as mean ± SD or number of subjects (percentage).

*BMI* body mass index, *FVC* forced vital capacity, *FEV*_*1*_ forced expiratory volume in 1 s, *IVC* inspiratory vital capacity, *IVC*_*fm*_ inspiratory vital capacity from the flowmeter.

Significant differences with the hEDS group: ***P < 0.001.

Concerning the subjects’ feeling of skill in lung volume perception, 10 participants in the hEDS group reported “doing an activity requiring good breathing control” compared to 9 in the control group. The activities were mostly physical. For patients, they were yoga, cycling, swimming, walking, horse riding, and dancing, and for control subjects, running, bodybuilding, judo, pilates, gymnastics, and cycling. Only one control subject cited sophrology as a non-physical activity requiring good ventilatory control. Only three participants (two hEDS, one control) answered “Yes” to the question “Would you be able to estimate the quantity/volume of air entering your lungs?” and when participants rated their ability to perceive on a numeric scale, the mean score obtained was 3.1 ± 2.5 for the hEDS group and 3.4 ± 2.5 for the control group, with no significant difference between the two groups.

Regarding spirometry, there was no significant difference between the two groups. IVC and IVC_fm_ measurements were strongly correlated (*r* = 0.96; *P* < 0.001).

### Lung volume perception

Regarding the IV_30_, there were significant differences between the hEDS and the control groups for the mean ΔIV in mL (*P* = 0.002) and in percentage (*P* = 0.001). There was no significant difference in the IV_50_ between the two groups (Table 2).

**Table 2:** Comparison of measurements of the accuracy of lung volume perception.

			hEDS	Control
IVC_fm_ (mL)			3227 ± 785	3607 ± 368
Mean ΔIV	IV_30_	(mL)	210 ± 124	140 ± 42**
		(%IV)	22.3 ± 14.6	13.1 ± 4.3**
	IV_50_	(mL)	214 ± 132	196 ± 155
		(%IV)	14.4 ± 10.6	10.8 ± 7.7

Data are presented as mean ± SD.

*ΔIV* absolute difference between the trial and the IV, *hEDS* hypermobile Ehlers-Danlos syndrome, *IVC*_*fm*_ inspiratory vital capacity from the lung volume perception device, *IV* target inspiratory volume.

Significant differences with the hEDS group: **P < 0.01.

### Ventilatory response to cognitive task

In the free condition (breathing without instruction), there was a significant difference between the hEDS and the control groups for f_B_ (*P* = 0.032). There was no other significant difference between the two groups in this condition (Table 3).

**Table 3:** Comparison of temporal and spatial variability of the ventilation during a free condition or a cognitive task between the hEDS and the control groups.

	Free condition		Cognitive task	
	hEDS	Control	hEDS	Control
Spatial				
V_T_ (mL)	647 ± 217	756 ± 222	708 ± 231	729 ± 215
V_T,SD_ (mL)	98 ± 83	99 ± 45	195 ± 107	124 ± 50*
CoefVar_V	0.147 ± 0.069	0.140 ± 0.078	0.278 ± 0.128	0.168 ± 0.071**
Temporal				
f_B_ (#.min^−1^)	14.7 ± 4.7	11.6 ± 4.1*	13.4 ± 5.2	13.6 ± 4.4
PP_SD_ (ms)	412 ± 180	495 ± 396	1332 ± 1054	697 ± 451*
CoefVar_t	0.093 ± 0.033	0.090 ± 0.066	0.243 ± 0.101	0.145 ± 0.096**
RMSSD (ms)	4592 ± 1587	5768 ± 1992	5290 ± 2359	4980 ± 1813
V̇_E_ (mL min^−1^)	8803 ± 2119	8222 ± 1894	8562 ± 2131	9317 ± 2328

Data are presented as mean ± SD.

*CoefVar_t* coefficient of variability for temporal domain, *CoefVar_V* coefficient of variability for spatial domain, *hEDS* hypermobile Ehlers-Danlos syndrome, *PP*_*SD*_ standard deviation of the time interval between each inspiratory peak, *RMSSD* root mean square of the time interval between each inspiratory peak, *f*_*B*_ breathing frequency, *IV* inspired volume, *IV*_*SD*_ standard deviation of the inspired volume, *V̇*_*E*_ minute ventilation, *V*_*T*_ tidal volume.

Significant differences with the hEDS group: *P < 0.05; **P < 0.01.

When performing the cognitive task, there were significant differences between the hEDS and the control groups for V_T,SD_ (*P* = 0.023), CoefVar_V (*P* = 0.004), PP_SD_ (*P* = 0.011) and CoefVar_t (*P* = 0.003).

**Figure 2:**
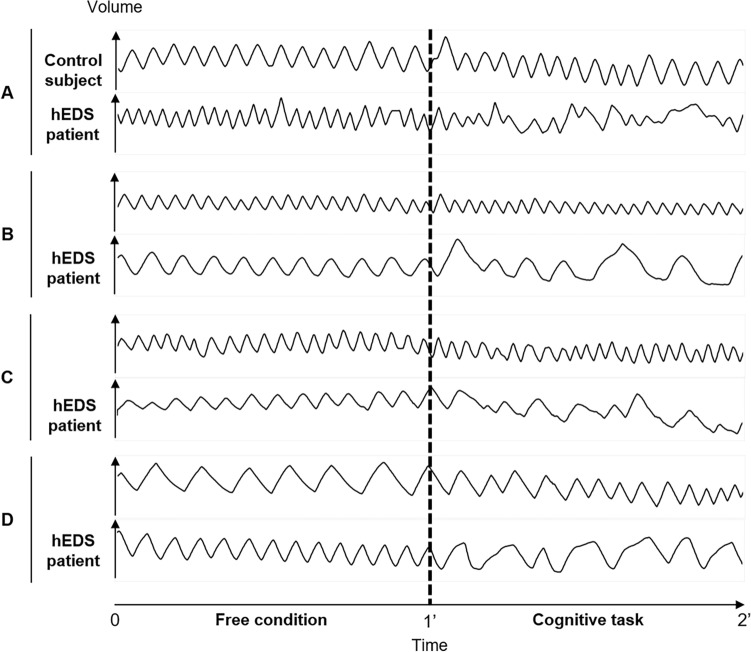
Examples of curves obtained during measurements of the effect of a cognitive task on ventilation. The left part of the curve corresponds to one minute of breathing without instruction; the right part corresponds to one minute of breathing performing the cognitive task. The curves are grouped by matched participants’ pairs (**A**–**D**). *hEDS* hypermobile Ehlers-Danlos syndrome.

Figure 2 illustrates, among hEDS patients, an erratic respiratory pattern (apnea, volume variations, and flow variations) during the cognitive task.

## Discussion

The objectives of this study were: (1) design a tool for the measure of lung volume perception; (2) investigate whether lung volume perception is impaired in hEDS patients; (3) investigate whether the control of breathing during a cognitive task is impaired in hEDS patients.

The tool designed to measure lung volume perception seems reliable, as indicated by the significant correlation (*r* = 0.96; *P* < 0.001) with the spirometer measurements. The results showed that the accuracy of lung volume perception was significantly reduced at 30% of IVC_fm_ in patients with hEDS and that they showed erratic ventilation when performing a cognitive task.

To our knowledge, this study is the first to explore the impact of a proprioceptive deficit on ventilation in hEDS. There was a significant difference between the two groups for IV_30_, with an average error of 22.3% in hEDS compared with 13.1% in healthy subjects, and similar results when expressed in mL. These results indicate that subjects with hEDS are less precise in perceiving their lung volume at moderate volumes. Impairment of this information could lead to abnormalities in ventilation regulation and potentially explain some of the dyspnea described in hEDS.

On the other hand, there is no significant difference for IV_50_, which suggests that both groups are more accurate at IV_50_. One hypothesis is that there would be more sensory information at IV_50_ than at IV_30_ due to an increased stretch of the different structures involved. One of the hypotheses to explain the proprioceptive deficit in hEDS subjects is that the structures containing the proprioceptive receptors would be more flexible in these subjects. Therefore, greater stretching might be necessary to trigger the nerve signal^16^. This is consistent with our results, since some signals would only occur at larger lung volumes in hEDS subjects. There is a need to further investigate the methodology of this measurement technique, such as looking for differences in healthy subjects at several lung volumes.

More generally, and to our knowledge, this is the first study to use a protocol inspired by joint proprioception to measure the accuracy of lung volume perception. Although this protocol seemed to be particularly adapted in the case of patients with hEDS, it would be interesting for future studies to establish standards and to investigate this lung volume perception in other respiratory pathologies such as chronic obstructive pulmonary disease or hyperventilation, but also in different physical activities. This tool could also be used to work on the perception of lung volume or to evaluate certain management techniques focused on ventilatory work.

Regarding the results of the ventilatory response to a cognitive task, an increase in temporal and spatial variability was observed in both groups during the cognitive task. However, this increase was more important in the hEDS group, which is responsible for the significant differences between the groups observed during the cognitive task but not during the free condition. This is seen in the visual analysis of the ventilatory curves (Fig. 2).

The increased variability of ventilatory spatial and temporal parameters during the addition of a cognitive task in the hEDS group could represent erratic ventilation during daily activities, such as difficulties in walking and balance when adding a cognitive task, as already mentioned in the literature^18,19^. However, this is the first time such difficulties in relation to ventilation are described.

As suggested in the first part of this study, a reduction in proprioceptive inputs from the musculoskeletal system involved in ventilation could cause abnormalities in ventilatory control. If we consider that the central nervous system also integrates these proprioceptive inputs to regulate ventilation, absent or erroneous information could lead to central dysregulation of ventilation. The addition of a cognitive task, which should normally produce minor changes in ventilation, would, therefore, result in the genesis of erratic ventilation due to dysregulation of ventilation control by anomalies in proprioceptive inputs from the musculoskeletal system involved in ventilation.

On visual analysis of the curves, some apnea intervals can be observed (Fig. 2, panel B, hEDS subject). It would be relevant to explore the link between these and the respiratory blockages described in hEDS by some authors^8,27^.

The f_B_ was found to be higher in the free condition in the hEDS group than in the control group. This is consistent with our clinical observations of a tendency for hyperventilation in patients with hEDS. The question of the energetic cost of faster ventilation may also arise, especially in a pathology frequently associated with chronic fatigue^28^.

Considering that physical activity requires more conscious joint control in subjects with hEDS due to increased laxity, this control could be viewed as a cognitive task and lead to ventilatory difficulties during physical activity. Erratic ventilation during physical activity could be a factor in exercise intolerance, and the altered lung volume perception could be one explanation for the dynamic hyperinflation described in these patients in a previous study^29^. These data open up new therapeutic perspectives in the management of respiratory disorders in hEDS.

One limitation of the study is the moderate number of subjects included. A larger study would be required to confirm these results. Although the hypothesis that has been proposed was oriented towards an influence of sensory inputs coming from the musculoskeletal system of the thoracic cage it is not possible to exclude a sensory influence from upper airway. The relative influence of each other could be the subject of a future study.

## Conclusions

This study demonstrates an impaired perception of lung volume at lower percentages of IVC and an increased difficulty in regulating ventilation during a cognitive task in patients with hEDS compared to healthy subjects. These elements may help to understand the respiratory manifestations found in hEDS.

## Data Availability

The data that support the findings of this study are available from the corresponding author upon reasonable request.

## Abbreviations

ΔIV: Absolute difference between the trial and the IV
CoefVar_t: Temporal coefficients of variability
CoefVar_V: Spatial coefficients of variability
EDS: Ehlers-Danlos syndrome
f_B_: Breathing frequency
FEV_1_: Forced expiratory volume in one second
FVC: Forced vital capacity
hEDS: Hypermobile Ehlers-Danlos syndrome
IV_x_: Inspiratory volume at x% of IVC_fm_
IVC: Inspiratory vital capacity (spirometer)
IVC_fm_: Inspiratory vital capacity with flowmeter
PP: Time interval between two inspiratory peaks
RMSSD: Root mean square of PP
V̇_E_: Minute ventilation
V_T_: Tidal volume
